# 10227 A Framework for Bringing Secondary Analysis of EHR Data to Geographically Dispersed Clinician Scientists

**DOI:** 10.1017/cts.2021.527

**Published:** 2021-03-30

**Authors:** James McClay, Jerrod Anzalone, Carol Geary, Ying Zhang

**Affiliations:** University of Nebraska Medical Center

## Abstract

ABSTRACT IMPACT: The described framework will enable other sites with a well-defined apparatus for enabling the secondary analysis of EHR data for research through education, team science, and resource consolidation. OBJECTIVES/GOALS: EHR’s potential to improve healthcare outcomes extends far beyond the clinic. This vast repository of clinical insights has dramatic potential for biomedical research. To enhance accessibility for busy clinicians and underserved populations, we describe a framework for interfacing with EHR locally and through national network participation. METHODS/STUDY POPULATION: The Institutional Development Award (IDeA) program, which began in 1993, broadens NIH funding’s geographic distribution for biomedical research. Included in this is the IDeA Networks for Clinical and Translational Research, which focuses on enhancing clinical and translational science across a network of IDeA-states with traditionally underserved communities and rural providers. A prior survey of the needs and capabilities of IDeA-CTR centers identified the need for improved research support. Based on our annual member survey we developed a process for supporting distributed research projects across the GP-CTR. NIH also recently made a funding announcement for the IDeA-CTR community identifying EHR research as a major priority in responding to the COVID-19 pandemic. RESULTS/ANTICIPATED RESULTS: Results from site interviews and member surveys show a clear need for dedicated resources to navigate the process of EHR-derived research. Most described a different set of requirements for increasing accessibility to EHR for research and a strong desire to participate in research networks. Local investigators cited a lack of tools, educational materials, and accessibility. Initial efforts demonstrate strong research questions but limited technical, statistical, and terminological capabilities to succeed. In response, a pipeline for team science and promotion of projects from local phenotypes to national studies. We created a facilitator training program to expand the number of facilitators (n=22), quarterly training for investigators (n=104), and ongoing efforts to advance COVID-19 research. DISCUSSION/SIGNIFICANCE OF FINDINGS: As evidenced in the expanding number of EHR-based research networks there is a need for a system to promote project development and best practices. The proposed model promotes education, resource sharing, and team formation to advance clinical questions from the idea stage toward national research network participation.

